# Comparative X-ray Diffraction Analysis of the Hydroxyapatite Crystal Structure in Developing and Mature Lumbar Vertebrae

**DOI:** 10.7759/cureus.84669

**Published:** 2025-05-23

**Authors:** Volodymyr Mavrych, Olena Bolgova, Abdalrahman Abuzubida

**Affiliations:** 1 College of Medicine, Alfaisal University, Riyadh, SAU

**Keywords:** biomineralization, bone development, crystallography, hydroxyapatite, vertebrae, x-ray diffraction, xrd

## Abstract

The ultrastructural characteristics of bone mineral composition during vertebral development remain incompletely understood. This study investigated the crystallographic parameters of hydroxyapatite in L3 vertebrae, comparing samples from newborn (n = 12) and adult (n = 12) specimens. X-ray diffraction analysis was performed using a DRON-3 diffractometer with copper Kα radiation (λ = 0.15433 nm) operating at 30 kV and 20 mA. Diffraction patterns were recorded from 2° to 37° at 1°/min, focusing on the characteristic hydroxyapatite peaks in the 30-34° range. Unit cell parameters were calculated using the (211), (112), and (300) reflections, while crystallite sizes were determined using the Scherrer method. Results revealed significant differences in crystallographic parameters between age groups, with adult samples showing larger crystallite sizes (18.12 ± 0.87 nm vs. 8.92 ± 0.25 nm, p < 0.001) and more defined diffraction peaks. The hexagonal unit cell parameters also differed significantly, with mature bone exhibiting increased lattice parameters in both the a-axis (9.451 ± 0.014 Å vs. 9.391 ± 0.014 Å, p < 0.001) and c-axis length (6.891 ± 0.010 Å vs. 6.860 ± 0.017 Å, p < 0.001) compared to developing bone. While adult specimens showed trends toward greater crystallinity with lower amorphous calcium (-10.76 wt%) and higher crystalline calcium (+2.60 wt%), these differences did not reach statistical significance. These findings provide new insights into the maturation process of bone mineral structure and may contribute to understanding age-related changes in bone mechanical properties.

## Introduction

Bone tissue represents one of the most remarkable biological materials in nature, serving multiple critical functions, including structural support, protection of vital organs, mineral homeostasis, and hematopoiesis. The extraordinary mechanical properties of bone are derived from its composite structure consisting of an organic matrix (primarily type I collagen) reinforced by inorganic mineral crystals. This mineral phase, comprising approximately 65-70% of mature bone tissue by weight, consists predominantly of hydroxyapatite with the chemical formula Ca₁₀(PO₄)₆(OH)₂ [1]. The precise arrangement, size, morphology, and crystallographic properties of these hydroxyapatite crystals significantly influence the mechanical and biological properties of bone tissue throughout development [2,3]. As the skeleton develops, it is not just the size and shape of the bones that change; there are also tiny, nanoscale changes happening in the structure of the mineral crystals that make up bone [4]. These subtle changes play a big role in how bones become stronger and better suited to handle physical stress. Understanding them can also help us make sense of certain bone conditions and how they develop over time [5].

The vertebral column, particularly the lumbar region, bears substantial mechanical loads and undergoes significant structural changes during growth and development [6]. Despite its biomechanical importance, detailed characterization of age-related changes in hydroxyapatite crystal parameters within vertebral bone remains incomplete [7]. While previous research has investigated age-related changes in long bones and cranial bones, comprehensive crystallographic studies focusing specifically on vertebral development are relatively sparse in the scientific literature [8].

X-ray diffraction (XRD) is a widely used and highly effective technique for examining the structural characteristics of bone mineral. As a nondestructive method, it allows researchers to gather detailed information about crystal size, orientation, lattice structure, and overall crystallinity without altering the sample [9]. By analyzing the unique diffraction patterns generated when X-rays interact with the crystalline components of bone, particularly hydroxyapatite, scientists can quantitatively assess how these mineral crystals are organized within the collagen matrix [10].

Unlike the rest of the bones in the body, the vertebral column follows a distinct developmental path, shaped by its unique mechanical demands and growth patterns [11]. The lumbar vertebrae, in particular, are subject to complex, multidirectional forces and continue to remodel significantly over a lifetime [12]. Studying the nanoscale mineral characteristics of vertebral bone at different stages of development can offer valuable insights into how the spine adapts to mechanical stress during growth and maturation [13].

The development of hydroxyapatite crystals in bone is influenced by several key factors. The collagen matrix provides the initial framework, acting as a template where tiny mineral crystals begin to form, specifically within the small gaps between collagen molecules [14]. Beyond collagen, non-collagenous proteins like osteocalcin, osteopontin, and bone sialoprotein play essential roles in guiding where and how these crystals form, grow, and align [15]. As the mineral phase matures, it undergoes further changes, including transformations in crystal structure, size, and chemical composition, all of which are critical to the overall strength and function of bone [16].

In the early stages of bone development, mineral deposits primarily consist of amorphous calcium phosphate and poor crystalline hydroxyapatite. As the bone matures, these initial phases gradually convert into more well-ordered hydroxyapatite, characterized by larger crystal size and reduced lattice strain [17]. This transformation plays a vital role in shaping the mechanical properties of bone, influencing its stiffness, strength, and resistance to fracture [18]. By quantitatively analyzing hydroxyapatite crystal features at various stages of development, researchers can gain important insights into how bone structure relates to its function over time.

In bone mineral research, several key crystallographic parameters are commonly assessed to understand the structure of hydroxyapatite. These include crystallite size (typically estimated using the Scherrer equation based on X-ray peak broadening), lattice parameters (specifically the a-axis and c-axis dimensions of the unit cell), and the crystallinity index, which reflects how structurally ordered the crystal lattice is [18]. These measurements are obtained by carefully analyzing XRD patterns, with particular attention to the hydroxyapatite peaks between 30° and 34° in the 2θ range, which correspond to the (211), (112), and (300) crystallographic planes [10,18].

Multiple studies have highlighted age-related changes in the crystallographic properties of bone mineral. For instance, Handschin and Stern [10] reported a steady increase in both crystal size and crystallinity of hydroxyapatite in human femoral cortical bone from childhood through old age. Similarly, Boskey et al. [19] observed shifts in crystal size and structural perfection in murine bone, both during normal development and in various genetic models of skeletal disease. However, these trends can differ depending on the specific bone being studied, as local mechanical forces and biological factors significantly influence mineral characteristics [20].

Understanding the normal developmental patterns of bone mineral crystallography carries important clinical significance. Changes in the properties of hydroxyapatite crystals have been linked to a range of skeletal disorders, such as osteoporosis, osteogenesis imperfecta, and rickets [21]. By establishing reference data that capture how bone mineral crystallography typically evolves during growth, researchers can more effectively detect and analyze abnormalities in bone mineralization associated with these conditions [22].

In recent years, advances in analytical techniques have allowed for a more comprehensive understanding of bone mineral properties. Methods such as Fourier transform infrared spectroscopy, transmission electron microscopy, and small-angle X-ray scattering have offered deeper insights into the composition, shape, and structural organization of hydroxyapatite crystals within bone tissue [5]. Despite these developments, conventional XRD remains a widely used and reliable method for quantitatively evaluating the crystallographic features of bone mineral [22].

Despite advancements in analytical techniques, there are still important gaps in our understanding of how bone mineral crystallography changes throughout development, especially within the vertebral column. This study aims to help fill those gaps by performing a comparative XRD analysis of the hydroxyapatite crystal structure in newborn and adult lumbar vertebrae. Focusing on the L3 vertebra, a region subject to significant mechanical loading, the research explores how the crystal structure of bone matures during vertebral development.

This study aimed to investigate the crystallographic parameters of hydroxyapatite in L3 vertebrae by comparing samples from newborn and adult specimens, with a specific focus on identifying age-related changes in crystal structure, lattice parameters, crystallite size, and the distribution between amorphous and crystalline calcium. By using XRD analysis, the research sought to characterize the maturation process of bone mineral structure during vertebral development and establish quantitative relationships between age and specific crystallographic properties of vertebral hydroxyapatite.

## Materials and methods

Study design

This comparative study analyzed the hydroxyapatite crystal structure in dry third lumbar (L3) vertebrae, obtained from the Anatomy Department of Luhansk State Medical University, Ukraine, bone collection. Samples were collected from the superior anterior part of the L3 vertebral bodies of 12 newborn and 12 adult skeletons. All samples were incinerated at 600°C for 12 hours to convert to ashes. The study focused on characterizing age-related differences in crystallographic parameters of vertebral bone minerals using XRD analysis. All specimens were processed into bone powder form for diffraction measurements. XRD analysis was utilized to analyze the physical properties of bone hydroxyapatite at the State Scientific-Research and Design Institute of Chemical Technologies “Khimtekhnologiya,” Siverskodonetsk, Ukraine.

Data collection

XRD measurements were performed using a DRON-3 diffractometer (Bourevestnik, Russia) with a GUR-5 goniometric attachment. The system operated with copper Kα radiation (λ = 0.15433 nm) at 30 kV voltage and 20 mA anode current. Diffraction patterns were recorded from 2° to 37° at a scanning rate of 1° per minute. The analysis focused on characteristic hydroxyapatite peaks in the 30-34° range, specifically examining the diffraction maxima corresponding to the (211), (112), and (300) planes with interplanar distances of d/n = 2.814, d/n = 2.779, and d/n = 2.721, respectively.

Data analysis

Crystallographic analysis consisted of several components: unit cell parameter calculations, crystallite size determination, and calcium distribution analysis. The hexagonal unit cell parameters were calculated using the following equation: 1/d² = 4/3[(h² + hk + k²)/a²] + l²/c², where d represents the interplanar spacing, h, k, and l are Miller indices, and a and c are lattice parameters of the unit cell [23].

Crystallite sizes were determined using the Scherrer method, applying the equation D = Kλ/(β cosθ), where K represents the Scherrer constant (0.9), λ is the X-ray wavelength, β is the peak broadening at half maximum intensity (full width at half maximum, FWHM), and θ is the Bragg angle [24,25]. We acknowledge that while the Scherrer method is widely used, it has certain limitations, including potential overestimation and inability to account for lattice strain effects. Peak characteristics analysis included measurement of the FWHM values and peak intensity ratios between adult and newborn samples for the three main diffraction peaks. Calcium content was assessed to determine the distribution between amorphous and crystalline forms in both age groups using peak-to-background ratio analysis [26].

Statistical comparison between newborn and adult groups was performed for all measured parameters using independent samples t-tests with R Statistics software. Statistical significance was set at p < 0.01. Quality control measures included instrumental broadening correction using a crystalline standard and validation of parameters through multiple reflection analysis.

## Results

**Figure 1:**
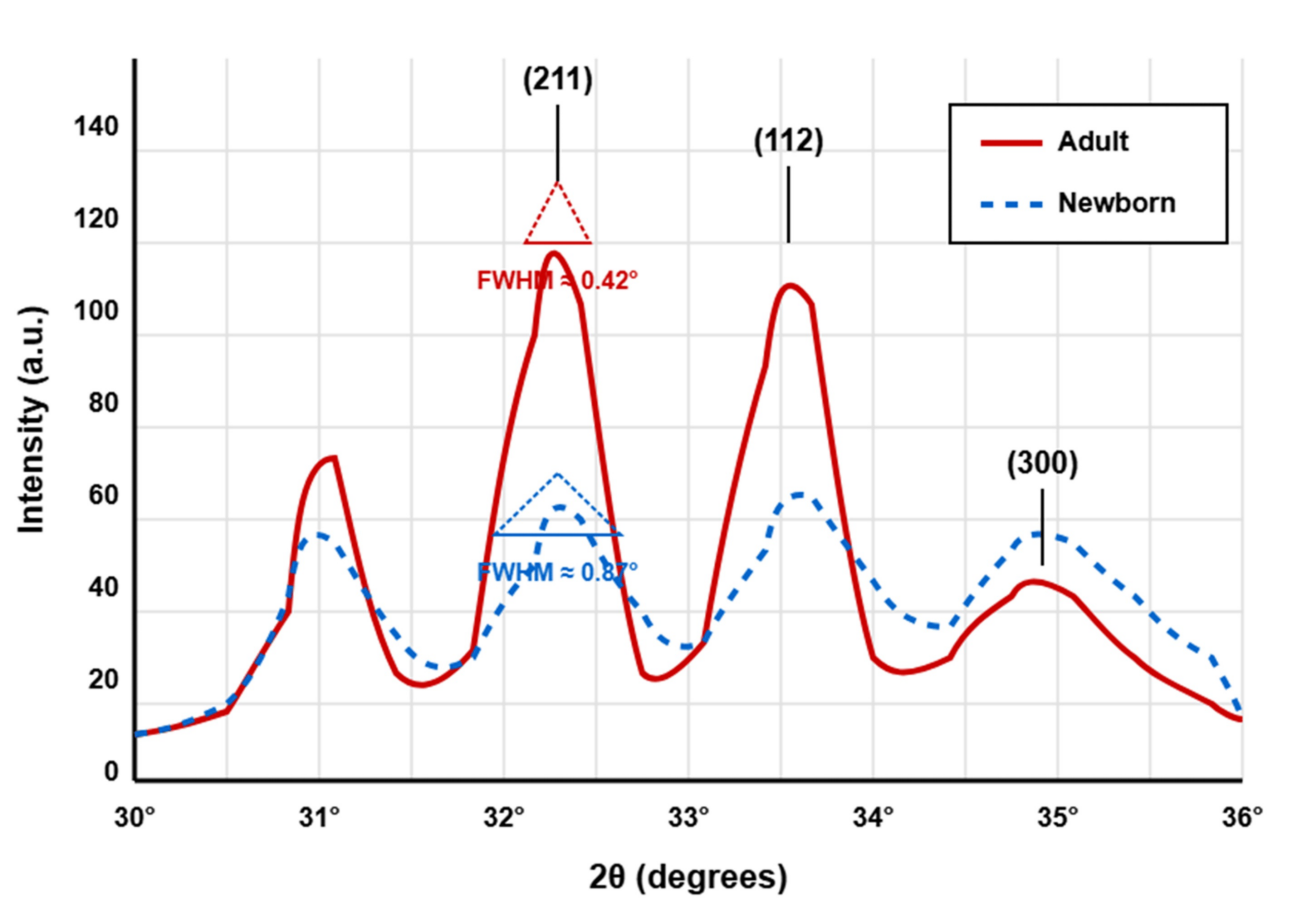
Representative XRD patterns of hydroxyapatite in L3 vertebrae from newborn (dashed blue line) and adult (solid red line) samples The main diffraction peaks corresponding to the (211), (112), and (300) planes are labeled. Note the increased peak intensity and reduced peak width (FWHM) in the adult pattern, indicating higher crystallinity and larger crystal size than the newborn sample. FWHM, full width at half maximum; XRD, X-ray diffraction

XRD analysis of hydroxyapatite in L3 vertebrae revealed significant structural differences between newborn and adult specimens. The characteristic diffraction patterns exhibited distinctive features in the 30-36° 2θ range, with the most prominent peaks corresponding to the (211), (112), and (300) crystallographic planes (Figure 1).

The adult samples demonstrated higher peak intensity and reduced peak width compared to newborn samples. This visual observation was confirmed by quantitative analysis of crystallographic parameters, which showed statistically significant differences between age groups for several key measurements (Table 1).

**Table 1:** Crystallographic parameters of hydroxyapatite in newborn and adult L3 vertebrae ^* ^Statistically significant difference (p < 0.001)

Parameter	Newborns (n = 12)	Adults (n = 12)	Difference (%)	p-value
Lattice parameter a (Å)	9.391 ± 0.014	9.451 ± 0.014	+0.64	p < 0.001^*^
Lattice parameter c (Å)	6.860 ± 0.017	6.891 ± 0.010	+0.45	p < 0.001^*^
a/c ratio	1.369 ± 0.002	1.372 ± 0.002	+0.19	p > 0.05
Unit-cell volume (Å³)	529.53 ± 1.65	534.29 ± 1.42	+0.90	p > 0.05

The hexagonal unit cell parameters showed expansion in both a-axis and c-axis dimensions in adult samples compared to newborn samples, with increases of 0.64% and 0.45%, respectively (p < 0.001). While the a/c ratio showed a slight increase in adult specimens (+0.19%), this difference did not reach statistical significance. Similarly, the unit-cell volume was larger in adult samples by 0.90%, but this difference was not statistically significant due to the relatively small magnitude of change despite the high precision of the measurements.

The most pronounced difference between age groups was observed in crystallite size, with adult samples exhibiting substantially larger crystals compared to newborn samples (Table 2). The average crystallite size was 103.2% greater in adult specimens, a highly significant difference (p < 0.001).

**Table 2:** Crystallite size and calcium distribution in newborn and adult L3 vertebrae ^*^ Statistically significant difference (p < 0.001)

Parameter	Newborns (n = 12)	Adults (n = 12)	Difference (%)	p-value
Average crystallite size (nm)	8.92 ± 0.25	18.12 ± 0.87	+103.2	p < 0.001^*^
Amorphous calcium (wt%)	12.38 ± 1.40	11.05 ± 1.78	-10.76	p > 0.05
Crystalline calcium (wt%)	70.00 ± 1.69	71.81 ± 3.18	+2.60	p > 0.05

The calcium content analysis suggested a shift toward greater crystallinity in adult specimens, with lower amorphous calcium (-10.76 wt%) and higher crystalline calcium (+2.60 wt%) compared to newborn samples. However, these differences did not reach statistical significance.

Detailed examination of the XRD peak characteristics provided additional evidence of structural differences between age groups (Table 3). The FWHM values were consistently smaller in adult samples across all measured peaks, indicating larger crystallite sizes according to the Scherrer equation. The FWHM ratio of approximately 2.0 between newborns and adults is consistent with the inverse relationship between FWHM and crystallite size in the Scherrer equation, supporting our finding of a 103.2% increase in crystallite size in adult samples. The peak intensity ratios (adult:newborn) were consistently greater than 1.0, demonstrating the higher degree of crystallinity in adult specimens.

**Table 3:** Comparison of XRD peak characteristics between age groups ^*^ FWHM: inversely related to crystallite size Values shown are mean ± SD across all samples. The narrower peak widths (smaller FWHM values) in adult samples indicate larger crystallite sizes, consistent with the Scherrer equation calculations presented in Table 2. FWHM, full width at half maximum; XRD, X-ray diffraction

Diffraction peak	Miller indices	Interplanar spacing (d/n, Å)	Peak intensity ratio (adult:newborn)	FWHM^*^ newborns (°2θ)	FWHM^*^ adults (°2θ)	FWHM ratio
Peak 1	-211	2.814	1.45 ± 0.12	0.87 ± 0.09	0.42 ± 0.04	2.07
Peak 2	-112	2.779	1.38 ± 0.09	0.92 ± 0.11	0.46 ± 0.05	2.00
Peak 3	-300	2.721	1.42 ± 0.14	0.89 ± 0.10	0.44 ± 0.05	2.02

In summary, the XRD analysis revealed significant age-related differences in hydroxyapatite crystal structure in L3 vertebrae. Adult samples demonstrated larger crystallite sizes, expanded lattice parameters, and a trend toward greater crystallinity compared to newborn samples. These structural differences were evident in both the visual appearance of the diffraction patterns and the quantitative analysis of crystallographic parameters.

## Discussion

The XRD analysis of hydroxyapatite in L3 vertebrae revealed significant structural differences between newborn and adult specimens, with findings that both align with and diverge from previous research on bone mineral crystallography.

Our study found adult vertebral samples exhibited substantially larger crystallite sizes compared to newborn samples, with a highly significant 103.2% difference (8.92 ± 0.25 nm in newborns vs. 18.12 ± 0.87 nm in adults, p < 0.001). These measured crystallite sizes are within the range typically reported in the literature for high-temperature treated bone samples (4-40 nm), which may be attributed to our sample preparation method involving incineration at 600°C [18]. Recent studies by Rabiei et al. have shown that incorporating multiple calculation methods, like Halder-Wagner, can yield more comprehensive size estimates [25]. The relatively larger crystallite sizes in our study compared to untreated bone samples are consistent with previous findings that heat treatment induces crystal growth, as reported by several researchers for samples prepared at 600°C [10,27]. Despite these methodological considerations, the significant increase in crystallite size with age observed in our study aligns with the findings of Handschin and Stern, who reported a steady increase in crystal size of human femoral cortical bone from childhood through old age [10].

The hexagonal unit cell parameters in our study showed expansion in both the a-axis (9.391 ± 0.014 Å in newborns vs. 9.451 ± 0.014 Å in adults) and c-axis dimensions (6.860 ± 0.017 Å in newborns vs. 6.891 ± 0.010 Å in adults) in adult samples compared to newborn samples, with statistically significant increases of 0.64% and 0.45%, respectively (p < 0.001). These lattice parameters are comparable to those reported by Wang et al. and demonstrate the maturation-related expansion of the hydroxyapatite crystal structure, possibly reflecting the incorporation of additional ions and the increased structural ordering that occurs during bone development [28].

While our study showed a trend toward greater crystallinity in adult specimens, with lower amorphous calcium (-10.76 wt%) and higher crystalline calcium (+2.60 wt%) compared to newborn samples, these differences did not reach statistical significance. This finding partially aligns with observations by Posner and Betts, who described the gradual conversion of amorphous calcium phosphate into more well-ordered hydroxyapatite during bone maturation [17]. The lack of statistical significance in our study may reflect the natural variability in mineral composition or methodological factors in calcium content analysis.

Our methodological approach using the Scherrer equation for crystallite size determination follows established protocols, although it differs from more recent studies that have incorporated multiple calculation methods to address the potential limitations associated with the Scherrer method. The Scherrer method is known to be affected by factors such as instrumental broadening and lattice strain, which can influence the final crystallite size estimates. Rabiei et al. and Kawsar et al. found that the Scherrer method consistently produces different size estimates compared to methods like Halder-Wagner, which may account for some differences between our absolute values and those reported in other studies [25,29].

The FWHM values in our diffraction patterns were consistently smaller in adult samples across all measured peaks (FWHM ratio of approximately 2.0), providing robust evidence for the age-related increase in crystallite size. This finding is consistent with studies by Boskey et al., who observed shifts in crystal size and structural perfection during normal development in murine bone [19].

Unlike studies that investigated the effects of varying synthesis conditions, such as pH and calcination temperature, on hydroxyapatite properties, our research focused on the natural development of bone mineral in vertebrae [28,29].

Limitations

This study is limited by our reliance on a single method for crystallite size determination. The Scherrer method, while widely used, does not account for peak broadening caused by lattice strain and instrumental factors, which we attempted to correct for using a crystalline standard. However, even with these corrections, some methodological constraints remain that could affect the accuracy of our size estimates. This methodological constraint makes direct comparisons with studies using different calculation approaches challenging and may affect the precision of the actual differences in crystal structure between age groups.

The sample preparation process involving incineration at 600°C for 12 hours introduces another significant limitation. While this temperature effectively removes organic material to facilitate XRD analysis, it may simultaneously alter aspects of the original crystal structure, including potential changes to crystallite size, crystallinity, and lattice parameters. This makes it difficult to differentiate between preparation-induced changes and natural biological differences in the bone mineral structure. Further, our sample demographics (n = 12 for each age group) represent only two distinct developmental stages without capturing intermediate phases of bone maturation.

## Conclusions

Our findings on the age-related changes in hydroxyapatite crystal structure in L3 vertebrae generally align with the broader literature on bone mineral development while providing specific data on vertebral bone that was previously lacking. The observed differences in crystallite size and lattice parameters between newborn and adult samples reflect the natural maturation process of bone mineral structure and provide valuable insights into the structural basis of age-related changes in bone mechanical properties. Future studies could benefit from incorporating multiple calculation methods for crystallite size determination and more detailed analysis of strain effects to further enhance our understanding of bone mineral crystallography during vertebral development.
